# Effect of Repetitive Glucose Spike and Hypoglycaemia on Atherosclerosis and Death Rate in Apo E-Deficient Mice

**DOI:** 10.1155/2015/406394

**Published:** 2015-08-20

**Authors:** Kenichi Nakajima, Tomoya Mita, Yusuke Osonoi, Kosuke Azuma, Toshiyuki Takasu, Yoshio Fujitani, Hirotaka Watada

**Affiliations:** ^1^Department of Metabolism & Endocrinology, Juntendo University Graduate School of Medicine, 2-1-1 Hongo, Bunkyo-ku, Tokyo 113-8421, Japan; ^2^Center for Molecular Diabetology, Juntendo University Graduate School of Medicine, 2-1-1 Hongo, Bunkyo-ku, Tokyo 113-8421, Japan; ^3^Drug Discovery Research, Astellas Pharma Inc., 21 Miyukigaoka, Tsukuba-shi, Ibaraki 305-8585, Japan; ^4^Center for Therapeutic Innovations in Diabetes, Juntendo University Graduate School of Medicine, 2-1-1 Hongo, Bunkyo-ku, Tokyo 113-8421, Japan; ^5^Sportology Center, Juntendo University Graduate School of Medicine, 2-1-1 Hongo, Bunkyo-ku, Tokyo 113-8421, Japan

## Abstract

Epidemiological data suggest that postprandial hyperglycaemia and hypoglycaemia are potential risk factors for cardiovascular disease. However, the effects of repetitive postprandial glucose spikes, repetitive hypoglycaemia, and their combination on the progression of atherosclerosis remain largely unknown. The present study investigated the effects of rapid rises and falls in glucose, and their combination, on the progression of atherosclerosis in apolipoprotein (apo) E-deficient mice. In this study, apo E-deficient mice with forced oral administration of glucose twice daily for 15 weeks were used as a model of repetitive postprandial glucose spikes, and apo E-deficient mice given an intraperitoneal injection of insulin once a week for 15 weeks were used as a model of repetitive hypoglycaemia. In addition, we established a model of both repetitive postprandial glucose spikes and hypoglycaemia by combining the above interventions. Atherosclerosis was evaluated in all mice by oil red O staining. Administration of ipragliflozin, a selective inhibitor of sodium-glucose cotransporter 2, in the mouse model of repetitive glucose spikes inhibited the progression of atherosclerosis, whereas long-term repetitive glucose spikes, repetitive hypoglycaemia, and their combination had no significant impact on atherosclerosis. However, repetitive hypoglycaemia was associated with poor survival rate. The results showed that repetitive hypoglycaemia reduces the survival rate without associated progression of atherosclerosis in apo E-deficient mice.

## 1. Introduction

Patients with type 2 diabetes mellitus (T2DM) are at high risk of developing cardiovascular disease (CVD), which is also the most frequent cause of death in these patients. Thus, one of the main goals of management of T2DM is to reduce the onset of CVD.

While hyperglycaemia is presumed to play a significant role in the progression of atherosclerosis, several epidemiological studies have suggested that postprandial hyperglycaemia* per se* is an independent risk factor for developing CVD [1, 2]. In this regard, we demonstrated previously that temporary hyperglycaemia induces monocyte adhesion to endothelial cells in the aorta of rats [3] and that repetitive glucose spikes enhance atherosclerotic lesions in apolipoprotein (apo) E-deficient mice [4]. In the second of these studies, the progression of atherosclerosis was attenuated by administration of an *α*-glucosidase inhibitor with the associated reduced amplitude of glucose spikes [4]. Furthermore, an* in vitro* study showed that intermittent treatment of high blood glucose levels increases apoptosis of endothelial cells by increasing oxidative stress [5]. Similarly, T2DM patients with glucose spikes had high oxidative stress level and endothelial dysfunction [6]. Although data on this topic remain controversial [7], the studies cited above suggest that glucose fluctuation could adversely affect the progression of atherosclerosis.

On the other hand, most clinical studies showed that reducing HbA1c levels had no beneficial effects on the incidence of CVD [8–10], possibly due to attenuation of the beneficial glucose-lowering effect by increased incidence of hypoglycaemic events. Indeed, a recent study reported that hypoglycaemia was associated with increased risk of cardiovascular events and all-cause mortality in insulin-treated patients with type 1 diabetes mellitus and T2DM [11]. While it is well known that hypoglycaemia affects cognition, mood, and consciousness, it has also profound effects on blood constituents [12, 13], inflammatory cytokine levels [14, 15], and coagulation and fibrinolysis factors [16, 17], all of which could potentially enhance the progression of atherosclerosis. Indeed, we found that repetitive hypoglycaemia induced monocyte adhesion to endothelial cells in the aorta [18] and enhanced neointima formation after vascular injury [19] in nonobese diabetic Goto-Kakizaki (GK) rats through a surge of sympathetic nerve activity.

The above studies investigated the effect of either rapid rises or falls in glucose levels on monocyte adhesion to endothelial cells or neointima formation after vascular injury. On the other hand, no convincing* in vivo* data exist about the combined effect of downward and upward spikes in circulating glucose using a mouse model of atherosclerosis. The present study investigated the effects of rapid rises and falls in glucose, and the combination thereof, on the progression of atherosclerosis in apo E-deficient mice.

## 2. Materials and Methods

### 2.1. Animal Experiments

**Figure 1:**
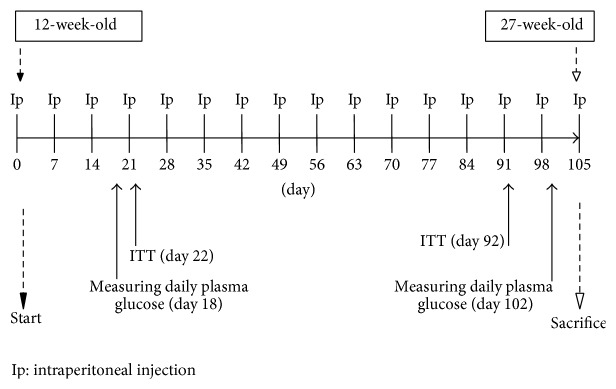
Experimental protocol. Mice of the control group (*n* = 22) were provided with water by oral gavage twice a day (9:00 AM and 4:00 PM) and received intraperitoneal injections of 10 mL/kg saline in the morning once a week. Mice of the glucose group (*n* = 22) were provided with glucose (2.0 g/kg) by oral gavage twice a day and received intraperitoneal injections of 10 mL/kg saline once a week. Mice of the glucose (2.0 g/kg) plus ipragliflozin group (*n* = 22) were provided with glucose (2.0 g/kg) twice a day and ipragliflozin (3 mg/kg) once a day by oral gavage and received intraperitoneal injections of 10 mL/kg saline once a week. Mice of the insulin group (*n* = 22) were provided with water by oral gavage twice a day and received intraperitoneal injections of 8 IU/kg insulin once a week. Mice of the glucose plus insulin group (*n* = 22) were provided with glucose (2.0 g/kg) by oral gavage twice a day and received intraperitoneal injections of 8 IU/kg insulin once a week.

The study protocol was reviewed and approved by the Animal Care and Use Committee of Juntendo University. Eight-week-old male apo E-deficient mice were purchased from Jackson Laboratory or Charles River Japan (Yokohama, Japan) and housed in specific pathogen-free barrier facilities at the Institute of Nihon Bioresearch Inc. (Gifu, Japan). Mice were maintained under a 12 h light/dark cycle and fed a standard rodent diet (CRF-1, Lot numbers 131008, 131203, and 140206, Oriental Yeast Co.). At 12 weeks of age, the apo E-deficient mice were divided into five treatment groups matched by body weight (BW) and plasma glucose level (Figure 1). Mice of the control group (*n* = 22) were provided with water by oral gavage twice a day (9:00 AM and 4:00 PM) and received intraperitoneal injections of 10 mL/kg saline in the morning once a week. Mice of the glucose group (*n* = 22) were provided with glucose (2.0 g/kg) by oral gavage twice a day and received intraperitoneal injections of 10 mL/kg saline once a week. Mice of the glucose (2.0 g/kg) plus ipragliflozin group (a sodium-glucose cotransporter 2 (SGLT2) selective inhibitor, Astellas Pharma Inc.; *n* = 22) were provided with glucose (2.0 g/kg) twice a day and ipragliflozin (3 mg/kg) once a day by oral gavage and received intraperitoneal injections of 10 mL/kg saline once a week. Mice of the insulin group (*n* = 22) were provided with water by oral gavage twice a day and received intraperitoneal injections of 8 IU/kg insulin once a week. Mice of the glucose plus insulin group (*n* = 22) were provided with glucose (2.0 g/kg) by oral gavage twice a day and received intraperitoneal injections of 8 IU/kg insulin once a week. Food intake and BW were measured weekly. At days 18 and 102, we recorded changes in plasma glucose and insulin concentrations in almost half of the mice after oral administration of saline or glucose or ipragliflozin while feeding normally. We also monitored changes in plasma glucose concentrations in almost half the remaining mice after insulin or saline injection at day 22 and day 92 while feeding normally. In those experiments, the mice in each group were further divided into two groups (*n* = 11, each) to reduce the burden of frequent blood sampling for measuring plasma glucose and insulin. All mice in all groups were sacrificed at 27 weeks of age to evaluate atherosclerotic changes.

### 2.2. Blood Testing

Plasma glucose levels were measured spectrophotometrically (U-3010, Hitachi High-Technologies Corporation), and plasma insulin levels (immunoreactive insulin, IRI) were measured by ELISA (Ultra Sensitive “PLUS” Mouse Insulin ELISA Kit, Morinaga Institute of Biological Science, Inc.) using a microplate reader (Powerscan HT, DS Pharma Biomedical Co.). Total cholesterol, high-density-lipoprotein cholesterol, low-density-lipoprotein cholesterol, and triglycerides were also measured with automated chemistry analyzer (AU 400, Beckman Coulter Biomedical K.K.) by enzyme method, direct measuring method, enzymatic assay, or glycerol blanking method, respectively. The levels of various serum cytokines, including tumour necrosis factor-*α*, interleukin-1*β*, and interleukin-6, were measured using a Multiplex kit (Merck Millipore) that uses nonmagnetic polystyrene bead-based luminex 200xPONENT technology (Merck Millipore).

### 2.3. Immunohistochemistry

After sacrifice with intraperitoneal sodium pentobarbital (1 mg/kg; Abbott Laboratories), the heart and aorta of all mice were flushed with normal saline followed by 10% buffered formalin, as described previously [20–22]. The aorta was excised from the root to the abdominal area, and then the connective and adipose tissues were removed from the aorta manually. For quantitative analysis of atherosclerotic lesions in the aortic sinus, the hearts were cut in half and the top half was embedded in optimal cutting temperature compound. Then, 4 *μ*m thick cross sections at 50 *μ*m intervals were prepared with a cryostat. Six consecutive sections were also taken sequentially from just above the aortic valve throughout the aortic sinus and allowed to dry at room temperature for 30 minutes, before staining with oil red O. In addition, whole aortas were also stained with oil red O. Histological images were analyzed by ImagePro Plus software. The lesion areas were calculated by dividing the oil red O-positive area by the total luminal area of the aorta.

### 2.4. Statistical Analysis

Results are presented as mean ± SEM of available data from surviving mice. Differences of laboratory blood test values among groups were examined by the Tukey* post hoc* or Student's *t*-test. Differences of histopathological parameters among groups were examined by Dunnett's multiple test or Student's *t*-test. The cumulative survival rates were compared by the log-rank test. A *P* value < 0.05 was considered significant. All tests were performed using the SAS software (SAS Institute, Japan).

## 3. Results

After 15 weeks of intervention, BW, food consumption, and lipid parameters were comparable among the five groups (Table 1).  Table 2 shows plasma glucose concentrations after oral administration of saline or glucose with or without ipragliflozin at 18 days and 102 days. As expected, the glucose group exhibited a significant increase in plasma glucose after the administration of glucose twice a day. In addition, the glucose plus insulin group showed a similarly acute rise in plasma glucose levels to those of the glucose group. Plasma glucose levels were comparable between the control group and the insulin group. Unexpectedly, the use of ipragliflozin significantly increased glucose levels before the first administration of glucose and caused only a modest decrease in plasma glucose levels after glucose administration.

Table 3 shows plasma insulin concentrations after oral administration of saline or glucose with or without ipragliflozin at 18 and 102 days. Increases in plasma insulin concentration in correspondence with glucose spikes were continuously observed in the glucose group, the glucose plus ipragliflozin group, and the glucose plus insulin group. Interestingly, glucose-induced insulin secretion tended to diminish by concomitant use of ipragliflozin.

At days 22 and 92, we evaluated the effects of insulin injection on plasma glucose levels (Table 4). As expected, insulin injection induced hypoglycaemia both in the insulin group and in the glucose plus insulin group, but not in the other three groups. However, mice of the glucose plus insulin group showed only modestly higher glucose levels at 92 days after repeated administration, compared with those measured at day 22. On the other hand, serum cytokine levels were not different among the groups (Table 1).

**Figure 2:**
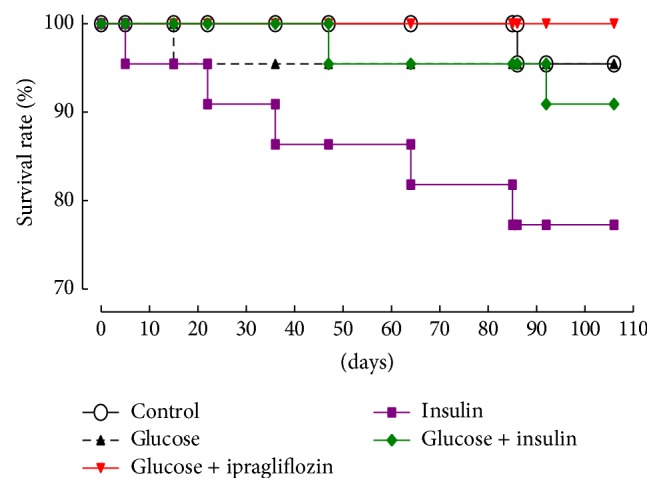
Survival rates of each treatment group. Kaplan-Meier curves indicated significantly reduced survival rates of mice of the insulin group. *P* < 0.05 by the log-rank test.

**Figure 3:**
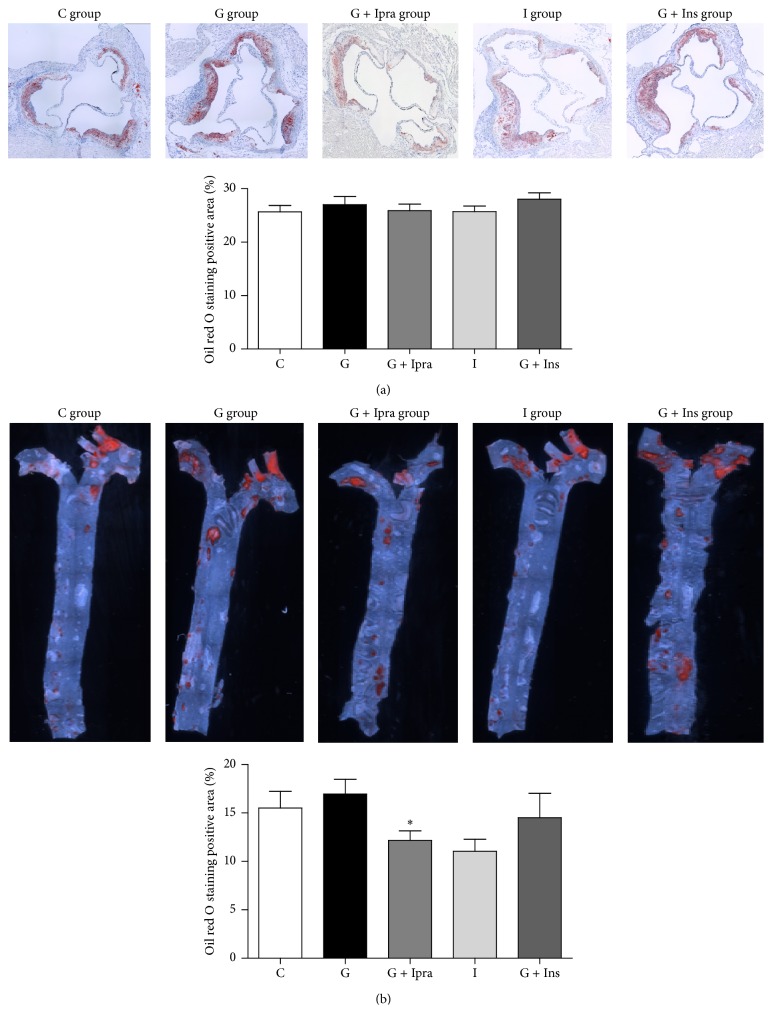
Atherosclerotic lesions in each treatment group. (a) Representative histological sections of aortic sinus stained with oil red O at 15 weeks after treatment. The area of oil red O-positive lesions in the aortic wall was evaluated. (b) Representative histological sections of the entire aorta stained with oil red O at 15 weeks after treatment. The area of oil red O-positive lesions in the entire aorta was evaluated. Data are mean ± SEM. ^*∗*^ *P* < 0.05 versus the glucose group by Student's *t*-test. C group: control group (*n* = 21), G group: glucose group (*n* = 20-21), G + Ipra group: glucose plus ipragliflozin group (*n* = 22), I group: insulin group (*n* = 16-17), and G + Ins group: glucose plus insulin group (*n* = 19-20).

Figure 2 shows the Kaplan-Meier curves, representing the survival rate of each group. Intriguingly, the survival rate was significantly lower in the insulin group compared with the other groups. To investigate the progression of atherosclerosis, we evaluated the oil red O-positive areas at the aortic valve level (Figure 3). The areas of oil red O staining of the aortic sinus were comparable among groups, although those of the glucose and glucose plus insulin groups tended to be slightly larger (Figure 3(a)). On the other hand, the oil red O-positive areas relative to the entire aorta of the glucose plus ipragliflozin group were significantly smaller than the glucose group (Figure 3(b)).

## 4. Discussion

In this study, repetitive hyperglycaemia, repetitive hypoglycaemia, and a combination of both did not enhance atherosclerosis in apo E-deficient mice, although repetitive hypoglycaemia increased the death rate. Coincidently, in our model, ipragliflozin suppressed atherosclerosis throughout the aorta of apo E-deficient mice with repetitive hyperglycaemia.

We reported previously that repeated increases in blood glucose induced by 5-week administration of maltose enhanced atherosclerosis in apo E-deficient mice [4], with peak levels of temporal hyperglycaemia that were similar to those recorded in the present study. However, long-term repeated increases in blood glucose levels had only a minor impact on atherosclerosis. It is possible that the glucose spike-induced atherosclerotic changes in our model might be eventually overwhelmed by concomitant high levels of cholesterol, although the underlying reasons for this effect remain unknown.

To our surprise in the present study, the atherosclerotic areas of the entire aorta were significantly smaller in the glucose plus ipragliflozin group compared with the glucose group. However, because the expression of SGLT2 in rodents is kidney-specific [23], any direct beneficial effects of SGLT2 inhibitors on the vasculature could not be anticipated, raising the possibility of other indirect effects of ipragliflozin on atherosclerosis. First, the inhibitory effects of ipragliflozin on atherosclerosis might simply reflect the glucose-lowering effect of this agent even though the dosage of ipragliflozin used in this study induced only a modest and almost nonsignificant reduction in glucose. Second, ipragliflozin tends to reduce glucose-induced temporal rises in insulin levels, because pharmacological inhibition of SGLT2 enhances urinary glucose excretion and thus reduces blood glucose levels independent of insulin actions [24]. Therefore, it is possible that improvement in temporal hyperinsulinaemia was associated with reductions in atherogenesis in our model. Although we demonstrated previously that temporal hyperglycaemia, but not temporal hyperinsulinaemia, induced monocyte adhesion to endothelial cells in rats [3, 25], temporal hyperinsulinaemia could probably indirectly promote atherosclerosis through unknown mechanism. Finally, SGLT-2 inhibitors also act as osmotic diuretics, resulting in lower blood pressure alongside the glucose-lowering effect [26]. These effects potentially contributed to the prevention of atherosclerosis observed herein.

On the other hand, random plasma glucose levels modestly but significantly increased from baseline on days 12 and 102, but not days 22 and 92, in the glucose plus ipragliflozin group. These factors may attenuate the favourable effect of ipragliflozin on the progression of atherosclerosis. Since the exact reasons for these unexpected results are largely unknown, evaluation of the possible metabolism-related factors, such as hepatic glucose production and plasma glucose levels, should be addressed in future studies.

Recent studies showed that hypoglycaemia is associated with increased mortality from CVD and other causes [11, 27, 28]. The Action to Control Cardiovascular Risk in Diabetes (ACCORD) study demonstrated a small but significant inverse relationship between the number of hypoglycaemic events and the risk of death in the intensive treatment group [27]. In the same study, higher HbA1c levels in the intensive treatment group were also associated with increased mortality [29]. On the other hand, the Action in Diabetes and Vascular Disease: Preterax and Diamicron Modified Release Controlled Evaluation (ADVANCE) showed that severe hypoglycaemia was also shown to be associated with increased mortality [28]. However, in the same study, the mortality rates were lower among subjects who experienced severe hypoglycaemia in the intensive treatment group than those in the standard group in that study, while severe hypoglycaemia was more frequent in the intensive treatment group [30]. Taking these results into consideration, it may be important to achieve appropriate glycaemic control without increased hypoglycaemic events in order to reduce mortality in the management of diabetes. In the present study, mice of the insulin group had the highest death rate, followed by those of the glucose plus insulin group. Because hypoglycaemia became milder at the end of our study in the glucose plus insulin group compared to the insulin group, the death rate was seemingly largely dependent on the severity of hypoglycaemia. However, the atherosclerotic lesions in the insulin group were not expanded, suggesting that hypoglycaemia-related death was not associated with events caused by advanced atherosclerosis, at least under the context of this experiment. On the other hand, we demonstrated previously that repetitive hypoglycaemia promoted the initial stage of atherosclerosis [18] and neointima formation after vascular injury in GK rats [19]. The exact reason for such discrepancy is not clear at present, but it could reflect differences in the stage of atherosclerosis, species, and the levels of cholesterol in diabetic and nondiabetic models.

What are the factors associated with death during hypoglycaemia? One possibility was raised by results showing that hypoglycaemia was associated with electrocardiographic changes, wherein severe hypoglycaemic events were significantly associated with prolonged corrected QT in patients with type 1 diabetes, reflecting abnormalities in ventricular myocardial repolarisation [31]. Notably, such arrhythmogenic effects that were induced, at least in part, by hypoglycaemia through the lowering of serum potassium or augmentation of sympathetic nerve activity could be life threatening. In addition, hypoglycaemia is known to induce platelet aggregation and inflammation [28], changes that could potentially increase plaque vulnerability without the associated progression of atherosclerosis. These findings emphasize the need for further experiments to address this issue, especially using mice models of plaque rapture [32].

The present study has certain limitations. First, we used higher dosage of insulin than those for the treatment of the patients with T2DM. The main reason was that those dosages were required in order to induce hypoglycaemia. Second, we did not measure serum and urinary levels of counter regulatory hormones during hypoglycaemia. Third, in this study, we evaluated only a few inflammatory cytokines. Thus, measurement of other cytokines is probably needed in the future studies. Forth, we could not evaluate the effect of insulin injection on proliferative potential [33].

In conclusion, our data suggested that long-term glucose fluctuations, including repetitive postprandial glucose spikes, repetitive hypoglycaemia, and combinations thereof, have little effect on atherogenesis. In addition, the low survival rate with repetitive hypoglycaemia is not necessarily associated with increased atherosclerotic plaques.

## Figures and Tables

**Table 1:** Body weight, food intake, and laboratory data for each group after 15 weeks' administration.

	Control group (*n* = 21)	Glucose group (*n* = 21)	Glucose + iplagliflozin group (*n* = 22)	Insulin group (*n* = 17)	Glucose + insulin group (*n* = 20)
Body weight (g)	25.6 ± 0.3	25.7 ± 0.3	25.3 ± 0.3	25.8 ± 0.5	25.5 ± 0.4
Food intake (g/day)	3.1 ± 0.1	3.0 ± 0.1	3.0 ± 0.1	3.0 ± 0.1	2.9 ± 0.1
HbA1c (%)	3.8 ± 0.0	3.8 ± 0.1	3.8 ± 0.0	3.8 ± 0.0	3.8 ± 0.0
Total cholesterol (mmol/L)	14.0 ± 0.4	13.5 ± 0.5	14.8 ± 0.5	14.2 ± 0.5	14.2 ± 0.6
HDL cholesterol (mmol/L)	0.3 ± 0.0	0.3 ± 0.0	0.3 ± 0.0	0.3 ± 0.0	0.3 ± 0.0
LDL cholesterol (mmol/L)	2.2 ± 0.1	2.2 ± 0.1	2.4 ± 0.1	2.2 ± 0.1	2.3 ± 0.1
Triglycerides (mmol/L)	0.9 ± 0.1	0.7 ± 0.1	0.9 ± 0.1	0.8 ± 0.1	0.9 ± 0.1
Tumour necrotic factor-*α* (pg/mL)	15.8 ± 1.0	15.6 ± 0.9	14.4 ± 0.5	14.0 ± 0.7	15.1 ± 0.9
Interleukin-1*β* (pg/mL)	32.0 ± 3.0	30.5 ± 1.9	27.1 ± 1.4	28.7 ± 0.8	30.5 ± 3.0
Interleukin-6 (pg/mL)	11.9 ± 1.3 (*n* = 20)	21.4 ± 6.9 (*n* = 20)	11.7 ± 1.3 (*n* = 21)	13.8 ± 2.4	12.9 ± 1.4

Data are mean ± SEM.

**Table 2:** Daily plasma glucose profile of each group.

	Control group	Glucose group	Glucose + ipragliflozin group	Insulin group	Glucose + insulin group
Day 18	(*n* = 11)	(*n* = 11)	(*n* = 11)	(*n* = 10)	(*n* = 11)
Glucose level (mmol/L) after first administration					
0 min	11.9 ± 0.5	11.7 ± 0.4	13.5 ± 0.3^*∗*#^	13.0 ± 0.3	12.9 ± 0.3
15 min	12.3 ± 0.5	18.0 ± 0.5^*∗*^	16.9 ± 0.4^*∗*^	14.3 ± 0.6^#†^	21.8 ± 0.8^*∗*#†¶^
30 min	13.7 ± 0.6	15.6 ± 0.7	16.1 ± 0.5	15.1 ± 0.4	18.9 ± 1.0^*∗*¶^
60 min	12.8 ± 0.7	12.4 ± 0.6	12.8 ± 0.4	13.5 ± 0.4	14.4 ± 0.5
120 min	10.6 ± 0.4	10.3 ± 0.4	10.0 ± 0.2	12.2 ± 0.3^*∗*#†^	11.9 ± 0.3^#†^
Glucose level (mmol/L) after second administration					
0 min	9.9 ± 0.4	10.4 ± 0.3	10.2 ± 0.4	11.0 ± 0.3	11.1 ± 0.2
15 min	10.5 ± 0.4	17.4 ± 0.4^*∗*^	15.4 ± 0.4^*∗*#^	11.2 ± 0.3^#†^	19.3 ± 0.8^*∗*†¶^
30 min	11.4 ± 0.5	13.6 ± 0.7	12.6 ± 0.7	12.7 ± 0.5	14.3 ± 1.0^*∗*^
60 min	10.5 ± 0.6	10.8 ± 0.3	9.7 ± 0.3	10.7 ± 0.4	11.6 ± 0.3
120 min	9.2 ± 0.5	8.5 ± 0.2	8.8 ± 0.6	10.0 ± 0.4	11.0 ± 0.2^*∗*#†^

Day 102	(*n* = 11)	(*n* = 11)	(*n* = 11)	(*n* = 8)	(*n* = 10)
Glucose level (mmol/L) after first administration					
0 min	10.3 ± 0.4	10.5 ± 0.5	12.3 ± 0.4^*∗*#^	11.2 ± 0.2	11.6 ± 0.4
15 min	10.4 ± 0.6	18.0 ± 0.4^*∗*^	16.0 ± 0.7^*∗*^	11.8 ± 0.2^#†^	17.9 ± 0.6^*∗*¶^
30 min	11.3 ± 0.5	15.1 ± 0.6^*∗*^	14.4 ± 0.7^*∗*^	13.2 ± 0.3	15.3 ± 0.5^*∗*^
60 min	10.8 ± 0.6	12.6 ± 0.6	11.8 ± 0.5	11.7 ± 0.3	13.0 ± 0.3^*∗*¶^
120 min	10.0 ± 0.2	9.6 ± 0.3	10.5 ± 0.4	10.9 ± 0.3	11.5 ± 0.2^*∗*#^
Glucose level (mmol/L) after second administration					
0 min	8.7 ± 0.3	8.8 ± 0.4	9.8 ± 0.3	10.8 ± 0.2^*∗*#^	10.2 ± 0.4^*∗*#^
15 min	10.4 ± 0.4	16.5 ± 0.5^*∗*^	15.2 ± 0.8^*∗*^	11.0 ± 0.4^#†^	18.1 ± 0.8^*∗*†¶^
30 min	11.3 ± 0.5	12.5 ± 0.6	12.7 ± 0.6	12.2 ± 0.4	14.4 ± 0.9^*∗*^
60 min	10.0 ± 0.4	10.9 ± 0.5	11.0 ± 0.5	10.8 ± 0.6	11.8 ± 0.5
120 min	9.2 ± 0.4	9.1 ± 0.3	9.5 ± 0.3	10.2 ± 0.3	10.6 ± 0.3^*∗*#^

Data are mean ± SEM. ^*∗*^ *P* < 0.05 versus control, ^#^ *P* < 0.05 versus glucose, ^†^ *P* < 0.05 versus ipragliflozin, and ^¶^ *P* < 0.05 versus insulin.

Mice were provided with water (control group, insulin group) or glucose (glucose group, glucose + ipragliflozin group, and glucose + insulin group) by oral gavage twice a day (9:00 AM: first administration and 4:00 PM: second administration).

**Table 3:** Daily plasma insulin profile of each group.

	Control group	Glucose group	Glucose + ipragliflozin group	Insulin group	Glucose + insulin group
Day 18	(*n* = 11)	(*n* = 11)	(*n* = 11)	(*n* = 10)	(*n* = 11)
Insulin level (pmol/L) after first administration					
0 min	167.1 ± 22.4	143.0 ± 19.0	105.1 ± 8.6	136.1 ± 12.1	93.0 ± 6.9^*∗*¶^
15 min	93.0 ± 10.3	291.2 ± 60.3^*∗*^	182.6 ± 20.7^*∗*^	65.5 ± 1.7^#†^	211.9 ± 19.0^*∗*¶^
30 min	87.9 ± 10.3	146.5 ± 36.2	103.4 ± 5.2	74.1 ± 8.6^#†^	105.1 ± 6.9^¶^
60 min	105.1 ± 12.1	91.3 ± 17.2	65.5 ± 5.2^*∗*^	77.5 ± 6.9	68.9 ± 5.2
120 min	117.2 ± 8.6	94.8 ± 17.2	74.1 ± 6.9^*∗*^	82.7 ± 8.6	87.9 ± 8.6
Insulin level (pmol/L) after second administration					
0 min	120.6 ± 17.2	108.5 ± 24.1	74.1 ± 5.2^*∗*^	89.6 ± 8.6	94.8 ± 8.6
15 min	89.6 ± 8.6	261.9 ± 34.5^*∗*^	215.4 ± 25.8^*∗*^	65.5 ± 6.9^#†^	256.7 ± 29.3^*∗*¶^
30 min	77.5 ± 6.9	113.7 ± 22.4	72.4 ± 3.4	75.8 ± 10.3	87.9 ± 10.3
60 min	77.5 ± 6.9	67.2 ± 10.3	46.5 ± 6.9^*∗*^	63.8 ± 6.9	58.6 ± 5.2
120 min	89.6 ± 12.1	60.3 ± 6.9	60.3 ± 8.6	65.5 ± 5.2	75.8 ± 8.6

Day 102	(*n* = 11)	(*n* = 11)	(*n* = 11)	(*n* = 8)	(*n* = 10)
Insulin level (pmol/L) after first administration					
0 min	163.7 ± 24.1	144.7 ± 15.5	144.7 ± 19.0	110.3 ± 15.5	96.5 ± 6.9
15 min	98.2 ± 10.3	291.2 ± 37.9^*∗*^	253.3 ± 24.1^*∗*^	81.0 ± 6.9^#†^	241.2 ± 19.0^*∗*¶^
30 min	86.2 ± 6.9	124.1 ± 12.1	117.2 ± 12.1	82.7 ± 6.9	112.0 ± 6.9
60 min	86.2 ± 8.6	103.4 ± 6.9	74.1 ± 5.2	87.9 ± 10.3	84.4 ± 6.9
120 min	99.9 ± 20.7	101.7 ± 6.9	82.7 ± 10.3	94.8 ± 10.3	84.4 ± 8.6
Insulin level (pmol/L) after second administration					
0 min	117.2 ± 19.0	124.1 ± 12.1	98.2 ± 10.3	130.9 ± 24.1	122.3 ± 15.5
15 min	99.9 ± 8.6	398.0 ± 24.1^*∗*^	274.0 ± 24.1^*∗*#^	84.4 ± 10.3^#†^	286.0 ± 29.3^*∗*¶^
30 min	103.4 ± 12.1	141.3 ± 19.0	106.8 ± 6.9	101.7 ± 10.3	134.4 ± 20.7
60 min	74.1 ± 6.9	101.7 ± 10.3	81.0 ± 8.6	84.4 ± 10.3	93.0 ± 8.6
120 min	79.3 ± 10.3	87.9 ± 8.6	77.5 ± 8.6	101.7 ± 10.3	91.3 ± 12.1

Data are mean ± SEM. ^*∗*^ *P* < 0.05 versus control, ^#^ *P* < 0.05 versus glucose, ^†^ *P* < 0.05 versus ipragliflozin, and ^¶^ *P* < 0.05 versus insulin. Mice were provided with water (control group, insulin group) or glucose (glucose group, glucose + ipragliflozin group, and glucose + insulin group) by oral gavage twice a day (9:00 AM: first administration and 4:00 PM: second administration).

**Table 4:** Plasma glucose levels after intraperitoneal injection of saline or insulin in each group.

	Control group	Glucose group	Glucose + ipragliflozin group	Insulin group	Glucose + insulin group
Day 22	(*n* = 11)	(*n* = 10)	(*n* = 11)	(*n* = 11)	(*n* = 11)
Glucose level (mmol/L) after administration					
0 min	9.4 ± 0.2	9.5 ± 0.2	10.2 ± 0.3	9.7 ± 0.2	10.0 ± 0.3
15 min	9.4 ± 0.4	17.2 ± 0.7^*∗*^	15.2 ± 0.5^*∗*^	5.5 ± 0.2^*∗*#†^	7.0 ± 0.2^*∗*#†¶^
30 min	11.6 ± 0.4	14.8 ± 1.0^*∗*^	14.6 ± 0.4^*∗*^	5.0 ± 0.2^*∗*#†^	5.1 ± 0.2^*∗*#†^
45 min	12.6 ± 0.6	13.0 ± 0.7	13.3 ± 0.4	4.8 ± 0.2^*∗*#†^	5.0 ± 0.2^*∗*#†^
60 min	12.5 ± 0.4	12.8 ± 0.8	13.0 ± 0.3	4.3 ± 0.3^*∗*#†^	4.4 ± 0.2^*∗*#†^
90 min	11.5 ± 0.3	11.9 ± 0.6	10.8 ± 0.3	3.5 ± 0.5^*∗*#†^	3.1 ± 0.2^*∗*#†^

Day 92	(*n* = 11)	(*n* = 10)	(*n* = 11)	(*n* = 9)	(*n* = 10)
Glucose level (mmol/L) after administration					
0 min	8.8 ± 0.1	9.1 ± 0.2	8.6 ± 0.3	8.9 ± 0.2	9.0 ± 0.2
15 min	10.2 ± 0.4	16.9 ± 0.9^*∗*^	14.2 ± 0.8^*∗*^	5.2 ± 0.2^*∗*#†^	8.7 ± 1.1^#†¶^
30 min	11.4 ± 0.4	14.7 ± 0.7^*∗*^	12.5 ± 0.8	4.7 ± 0.2^*∗*#†^	6.0 ± 0.7^*∗*#†^
45 min	12.6 ± 0.3	13.5 ± 0.7	12.0 ± 0.7	4.4 ± 0.2^*∗*#†^	5.9 ± 1.1^*∗*#†^
60 min	12.9 ± 0.6	14.2 ± 0.8	12.1 ± 0.7	3.8 ± 0.2^*∗*#†^	5.8 ± 1.3^#†^
90 min	11.6 ± 0.5	12.4 ± 0.6	10.2 ± 0.7	3.2 ± 0.6^*∗*#†^	4.5 ± 1.1^*∗*#†^

Data are mean ± SEM. ^*∗*^ *P* < 0.05 versus control, ^#^ *P* < 0.05 versus glucose, ^†^ *P* < 0.05 versus ipragliflozin, and ^¶^ *P* < 0.05 versus insulin.

Mice received intraperitoneal injections of 10 mL/kg saline (control group, glucose group, and glucose + ipragliflozin group) or injections of 8 IU/kg insulin (insulin group and glucose + insulin group).

## Abbreviations

ACCORD: Action to Control Cardiovascular Risk in Diabetes
ADVANCE: Action in Diabetes and Vascular Disease: Preterax and Diamicron Modified Release Controlled Evaluation
apo: Apolipoprotein
BW: Body weight
CVD: Cardiovascular disease
GK rat: Goto-Kakizaki rat
SGLT2: Sodium-glucose cotransporter 2
SMCs: Smooth muscle cells
T2DM: Type 2 diabetes mellitus.
